# Safety of hepatitis E vaccination for pregnancy: a post-hoc analysis of a randomized, double-blind, controlled phase 3 clinical trial

**DOI:** 10.1080/22221751.2023.2185456

**Published:** 2023-03-15

**Authors:** Guohua Zhong, Chunlan Zhuang, Xiaowen Hu, Qi Chen, Zhaofeng Bi, Xinhua Jia, Siying Peng, Yufei Li, Yue Huang, Qiufen Zhang, Ying Hong, Youlin Qiao, Yingying Su, Huirong Pan, Ting Wu, Lihui Wei, Shoujie Huang, Jun Zhang, Ningshao Xia

**Affiliations:** a State Key Laboratory of Molecular Vaccinology and Molecular Diagnostics, National Institute of Diagnostics and Vaccine Development in Infectious Diseases, Collaborative Innovation Center of Biologic Products, School of Public Health, Xiamen University, Xiamen, People’s Republic of China; b Xiang’an Biomedicine Laboratory, Xiamen, People’s Republic of China; c Xiamen Innovax Biotech Company, Xiamen, People’s Republic of China; d The Affiliated Drum Tower Hospital of Nanjing University Medical School, Nanjing, People’s Republic of China; e National Cancer Center, National Center for Cancer Clinical Research, the Cancer Institute, Chinese Academy of Medical Sciences/Peking Union Medical College, Beijing, People’s Republic of China; f Peking University People’s Hospital, Beijing, People’s Republic of China; g The Research Unit of Frontier Technology of Structural Vaccinology of Chinese Academy of Medical Sciences, Xiamen University, Xiamen, People’s Republic of China

**Keywords:** Hepatitis E vaccine, Hecolin, human papillomavirus vaccine, pregnancy, safety

## Abstract

Special attention has been paid to Hepatitis E (HE) prophylaxis for pregnant women due to poor prognosis of HE in this population. We conducted a post-hoc analysis based on the randomized, double-blind, HE vaccine (Hecolin)-controlled phase 3 clinical trial of human papillomavirus (HPV) vaccine (Cecolin) conducted in China. Eligible healthy women aged 18–45 years were randomly assigned to receive three doses of Cecolin or Hecolin and were followed up for 66 months. All the pregnancy-related events throughout the study period were closely followed up. The incidences of adverse events, pregnancy complications, and adverse pregnancy outcomes were analysed based on the vaccine group, maternal age, and interval between vaccination and pregnancy onset. During the study period, 1263 Hecolin receivers and 1260 Cecolin receivers reported 1684 and 1660 pregnancies, respectively. The participants in the two vaccine groups showed similar maternal and neonatal safety profiles, regardless of maternal age. Among the 140 women who were inadvertently vaccinated during pregnancy, the incidences of adverse reactions had no statistical difference between the two groups (31.8% vs 35.1%, *p* = 0.6782). The proximal exposure to HE vaccination was not associated with a significantly higher risk of abnormal foetal loss (OR 0.80, 95% CI 0.38–1.70) or neonatal abnormality (OR 2.46, 95% CI 0.74–8.18) than that to HPV vaccination, as did distal exposure. Significant difference was not noted between pregnancies with proximal and distal exposure to HE vaccination. Conclusively, HE vaccination during or shortly before pregnancy is not associated with increased risks for both the pregnant women and pregnancy outcomes.

## Introduction

Hepatitis E (HE) is a common viral hepatitis caused by the hepatitis E virus (HEV) infection. There are an estimated 20 million HEV infections worldwide every year, leading to approximately 3.3 million symptomatic cases and 44,000 deaths [1]. Although HEV infection is an acute and self-limited disease, progressions to fulminant hepatic failure and other severe consequences have been recorded in pregnant women [2,3]. In some high-prevalence areas, HEV infection has become a major contributor to maternal mortality and abnormal foetal loss [4]. A substantially higher maternal case fatality rate in pregnant women with HEV infection, varied between 3.2% and 70%, was shown in a meta-analysis [5]. Other adverse pregnancy outcomes, including low birth weight, stillbirth, preterm birth (<32 weeks), and intrauterine deaths, were also reported to be highly associated with HEV infection [6].

A recombinant HE vaccine (Hecolin; Xiamen Innovax, Xiamen, China), which has been licensed in 2011 and 2020 in China and Pakistan, separately, is the world’s first and only vaccine to prevent HEV infection. It has been demonstrated with good safety and an efficacy of 100% within 12 months after the third dose in a large-scale, randomized, double-blind, placebo-controlled, phase 3 trial [7]. The efficacy of 93.3% (95% confidence interval [CI] 78.6–97.9) in the per-protocol set was confirmed within 4.5 years of follow-up [8]. The highly efficacious HE vaccine may hold great promise for reducing the high mortality and multiple adverse outcomes associated with HEV infection in pregnant women. However, concerns on whether HE vaccination has a causal effect on adverse pregnancy outcomes warrant further investigation in the absence of rigorous data in clinical trials currently. A published post-hoc analysis with a limited sample size of inadvertent HE vaccinees during pregnancy in Hecolin’s phase 3 trial did not show any potential risk for foetus [9].

Therefore, with a view to further investigation of the potential risk posed by HE vaccination during or shortly before pregnancy, we conducted a post-hoc analysis of pregnancy outcomes based on a large-scale phase 3 clinical trial of an *Escherichia coli* (*E. coli*)-produced human papillomavirus (HPV) bivalent vaccine, in which the control group received Hecolin. Given that the current safety data for the HPV vaccine is reassuring [10], we compared the safety profile between HE vaccination and HPV vaccination for pregnancy in the absence of the unvaccinated control population.

## Materials and methods

### Study population

This post-hoc analysis was based on a multi-centre, double-blind, randomized, and controlled phase 3 trial (ClinicalTrials.gov: NCT01735006) of an *E. coli*-produced HPV bivalent (type 16 and 18) vaccine (Cecolin, Xiamen Innovax, Xiamen, China) in China as described previously [11]. In brief, eligible healthy women aged 18–45 years from five study sites were randomly assigned in a 1:1 ratio with stratification of age (18–26 and 27–45 years) to receive three doses of Cecolin or Hecolin at months 0, 1, and 6. The main exclusion criteria included immunodeficiency disorder, previous severe allergic reactions after vaccination, severe internal disease, pregnancy, and previous HPV vaccination. All the participants were followed up at least for 66 months according to the protocol.

The trial was approved by the Independent Ethics Committee. Written informed consent was obtained from each participant, and the study was done in accordance with the principles of the Declaration of Helsinki, the standards of Good Clinical Practice, and Chinese regulatory requirements.

### Vaccines

The control HE vaccine (Hecolin, Xiamen Innovax, Xiamen, China) contains 30 μg of the purified HEV239 particulate antigen adsorbed to 0.8 mg aluminium hydroxide suspended in 0.5 mL buffered saline [7]. Hecolin, which has been licensed in China in 2011 and Pakistan in 2020, is the world’s first and only vaccine to prevent HEV infection. The HPV vaccine (Cecolin, Xiamen Innovax, Xiamen, China) contains 40 μg of HPV-16 and 20 μg of HPV-18 L1 VLPs, suspended in 0.5 mL of buffered saline and 208 μg of aluminium adjuvant [12]. Cecolin has been licensed in China, Morocco, Nepal, Thailand, and Congo (DRC) during 2019–2022, and has been included in World Health Organization (WHO) prequalification list in 2021 [13]. The three-dose-regimen for both Hecolin and Cecolin was given intramuscularly at 0, 1, and 6 months.

### Safety monitoring

Participants were observed for 30 minutes after each vaccination for the occurrence of any immediate reactions. All the participants were trained to record any solicited and unsolicited adverse events, medication administration, and other vaccinations occurred within 30 days after vaccination on the diary card distributed. Serious adverse events (SAEs) occurring within the 66-month study period were collected regularly by safety assessors. Adverse reactions, defined as adverse events related to vaccination, were judged by investigators with physician qualification according to the implementation rules, as described previously [14].

All the participants in the trial should have negative urine pregnancy test results before each vaccination and were requested to avoid pregnancy within eight months after the first dose. Women with a positive test result should be suspended the vaccination and may continue two weeks after the end of pregnancy (delivery or miscarriage). All pregnancy events during the study period were recorded in detail through periodic telephone or in-person investigations. For pregnant women planning to deliver, regular telephone follow-up should be conducted at least once every three months. The last telephone survey should be conducted one month after the end of delivery.

### Pregnancy outcomes and complications

Gestational age was measured from the first day of the last menstrual period (LMP) according to the international consensus. If LMP was unknown or unclear, gestational age would be reported based on ultrasound examination and an estimated LMP was also calculated. Pregnancy has three trimesters: first trimester (0–13 weeks), second trimester (14–26 weeks), and third trimester (27–40 weeks). The due date was the date of the first day of LMP plus 40 weeks (280 days), and maternal age was defined as the age at the time of the due date. Advanced maternal age was defined as 35 years or older. Pregnancy outcomes included termination and delivery. Elective termination was defined as an intentional abortion by an artificial procedure and was not considered as an adverse pregnancy outcome [15]. Adverse pregnancy outcomes referred to abnormal foetal loss and neonatal abnormalities. The former included spontaneous abortion, stillbirth, and termination of pregnancy caused by maternal complications, while the latter included abnormal weight, preterm birth, low Apgar scores, congenital anomaly, and other neonatal complications. Spontaneous abortion was defined as the termination of pregnancy without human interference prior to 28 weeks of gestation [16]. Stillbirth was defined as an abnormal foetal loss at 28 weeks of gestation and beyond. Terminations of pregnancy due to complications with a clear diagnosis were classified as a separate section of abnormal foetal loss. As for neonatal abnormalities, neonates weighing less than 2.5 kg or more than 4.0 kg were considered as abnormal weight, and Apgar score of less than 8 was considered low score. Preterm birth is defined as a live baby born before the completion of a 37-week gestation [17]. Congenital anomalies comprise a wide range of abnormalities of body structure or function that are present at birth [18]. Pregnancy complications were any health problems that occurred during pregnancy, which might involve the mother’s health, the baby’s health, or both [19]. We focused on the occurrence of ectopic pregnancy, gestational hypertension, gestational diabetes, eclampsia, and preeclampsia because of being common and important markers of maternal health.

### Statistical analyses

All statistical comparisons were made between the HE vaccine group and the HPV vaccine group using Student’s T-test, Chi-Squared test, Fisher’s exact test, Wilcoxon test, or Cochran-Mantel-Haenszel test.

Characteristics of women who became pregnant and their pregnancy outcomes and complications were displayed. Women who inadvertently received HE vaccine during pregnancy were matched in a 1:2 ratio to those HE vaccinees who were nonpregnant throughout the study period. The matched factors included age at enrolment (no more than two years of difference), receiving the same HE vaccine doses and study site. The incidences of adverse events in participants vaccinated during pregnancy, as well as in matched nonpregnant women, were summarized.

Pregnancy-related adverse events were divided into three parts: abnormal foetal loss outcomes, neonatal abnormality outcomes, and pregnancy complications in focus. To measure the impact of exposure time for pregnancy on adverse pregnancy outcomes and pregnancy complications, exposure types were classified as proximal and distal based on the interval between exposure and pregnancy. Proximal exposure was defined as vaccination during pregnancy or the onset of pregnancy within 90 days post any dose. Odds ratios with 95% confidence intervals were estimated with the use of a logistic regression model, with presence/absence of adverse pregnancy events considered as the dependent variable and the type of vaccine received and maternal age used as independent variables. The primary comparisons included: (1) pregnant women with any HE vaccine exposure vs those with any HPV vaccine exposure; (2) pregnant women with proximal HE vaccine exposure vs those with proximal HPV vaccine exposure; (3) pregnant women with distal HE vaccine exposure vs those with distal HPV vaccine exposure. In addition, comparison of pregnant women having proximal exposure with those having distal exposure was drawn specifically in the HE vaccine group, using the exposure types and maternal age as independent variables. To avoid masking the acute effects of vaccine, we conducted a sensitivity analysis based on defining proximal exposure as vaccination during pregnancy or the onset of pregnancy within 30 days post any dose. All analyses were performed using the generalized estimation equation (GEE) method to account for possible correlations between pregnancies of the same mother. The dependent variable was binomial (presence/absence of adverse pregnancy events), and hence the logit link function was used in the GEE model.

Pregnancy complications and adverse events were coded using the Medical Dictionary for Regulatory Activities (MedDRA, version 20.0) developed by ICH. SAS version 9.4 was used for all the analyses. All reported *p*-values are two-sided with an *α* value of 0.05.

## Results

**Figure 1.**
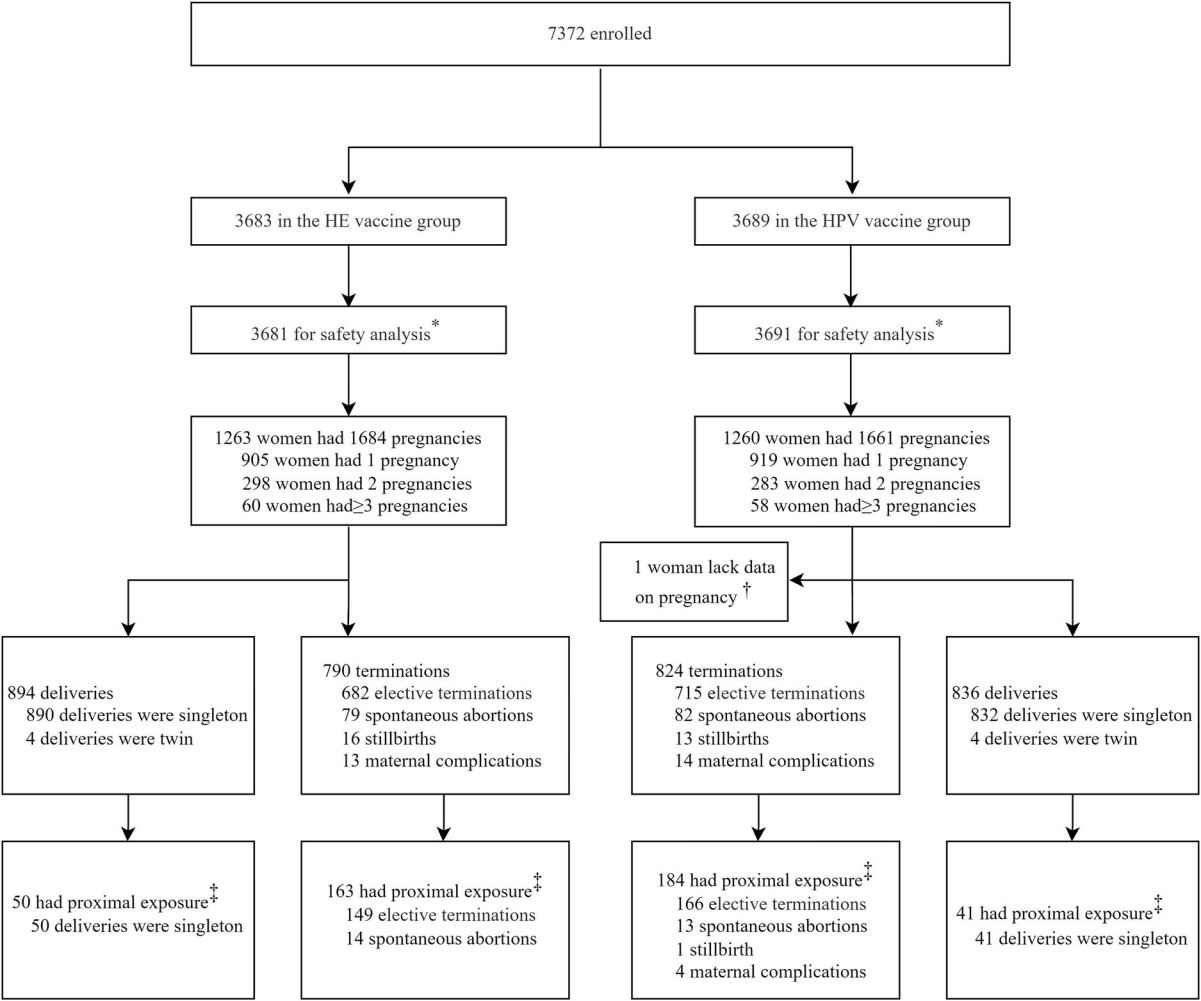
Study profile. HE: Hepatitis E. HPV: Human papillomavirus. All the women who received at least one dose of vaccine were followed up to 66 months. *By mistake, one participant in the HPV vaccine group was given the HE vaccine for dose 1, and two participants in the HE vaccine group were given the HPV vaccine for dose 3. These three participants were included in the HPV vaccine group for safety analysis, according to the protocol. ^†^One woman with one pregnancy event in the HPV vaccine group was excluded from the analysis due to loss of follow-up and incomplete information on pregnancy outcome. ^‡^Proximal exposure was defined as vaccination during pregnancy or the onset of pregnancy within 90 days post any dose. Abbreviations: HE, hepatitis E; HPV, human papillomavirus.

During 22 November 2012 to 1 April 2013, 7372 healthy women aged 18–45 years old were enrolled and randomly assigned to receive Hecolin (*n* = 3683) or the HPV vaccine (*n* = 3689) and were followed up until 1 August 2019 (Figure 1). During the follow-up period, 2523 (34.2%, 2523/7372) reported having at least one pregnancy, of which 1263 were in the HE vaccine group, and 1260 in the HPV vaccine group (Figure 1). One woman with one pregnancy event in the HPV vaccine group was excluded from the analysis because of incomplete information on pregnancy outcome. Baseline demographic characteristics of the two groups were generally comparable. The median follow-up time for the two groups was 68.5 and 68.2 months (*p* = 0.5947), respectively. 71.7% (905/1263) and 72.9% (918/1259) of the pregnant women had only one pregnancy in the two vaccine groups, respectively (Table 1). A total of 3344 pregnancy events occurred during the study, of which 1684 were in the HE vaccine group, and 1660 in the HPV vaccine group (Table 1). The median (*p* = 0.1094) and distribution (*p* = 0.0608) of maternal age were similar in two groups. The majority of pregnancies occurred after completing the full vaccination course, and the median interval between the onset of pregnancy and the last vaccination was 23.8 months and 22.7 months in the HE vaccine and HPV vaccine group (*p* = 0.2164). However, a total of 143 pregnancy events occurred in 140 pregnant women who were inadvertently vaccinated during pregnancy (66 in HE vaccine group and 77 in HPV vaccine group), possibly because early undetected pregnancy or the false negative test results. Only one woman in the HE vaccine group received more than one vaccination (two doses, first and second) during the same pregnancy event. The gestational stage of vaccine exposure was the first trimester for all pregnancy events with vaccination during pregnancy. The proportion of proximal exposure was in the minority in both groups (12.6% and 13.6%, *p* = 0.4376).

**Table 1.** Characteristics of HE or HPV vaccinees who became pregnant throughout the study.

Characteristic		HE vaccine group	HPV vaccine group	*p*-value
Pregnant women				
	No.	1263	1259	–
	Median follow-up months (IQR)	68.5 (66.6,69.4)	68.2 (66.6,69.3)	0.5947
	No. of pregnancy (%)			0.8479
	1	905 (71.7)	918 (72.9)	
	2	298 (23.6)	283 (22.5)	
	3	53 (4.2)	53 (4.2)	
	≥4	7 (0.6)	5 (0.4)	
Pregnancy events				
	No.	1684	1660	–
	Median maternal age (IQR)	28.0 (26.0, 30.0)	28.0 (26.0, 31.0)	0.1094
	Distribution of maternal age-No. (%)			0.0608
	<35	1505 (89.4)	1449 (87.3)	
	≥35	179 (10.6)	211 (12.7)	
	Site distribution-No. (%)			0.1087
	Liuzhou City	753 (44.7)	719 (43.3)	
	Funing County	388 (23.0)	446 (26.9)	
	Xinmi County	305 (18.1)	267 (16.1)	
	Fengning County	131 (7.8)	130 (7.8)	
	Yangcheng County	107 (6.4)	98 (5.9)	
	Vaccination during pregnancy*-No. (%)			0.3040
	Yes	66 (3.9)	77 (4.6)	
	No	1618 (96.1)	1583 (95.4)	
	Median interval months between the onset of pregnancy and the last vaccination (IQR)^†^	23.8 (10.8, 37.8)	22.7 (9.1 37.9)	0.2164
	Classification of vaccination exposure^‡^ -No. (%)			0.4376
	Proximal	213 (12.6)	225 (13.6)	
	Distal	1471 (87.4)	1435 (86.4)	

*Only one woman in HE vaccine group received more than one vaccination (two doses, first and second) during the same pregnancy event, and her stage of gestational vaccine exposure was defined as the stage at the time of the first dose. The gestational stage of vaccine exposure was the first trimester for all pregnancy events with vaccination during pregnancy.

^†^ Data were calculated only for those events without vaccine exposure during pregnancy.

^‡^ Proximal exposure was defined as vaccination during pregnancy or the onset of pregnancy within 90 days post any dose.

Abbreviations: HE, hepatitis E; HPV, human papillomavirus; No., number; IQR, interquartile range.

11.7% (390/3344) of pregnancy events were observed in women of advanced maternal age. As presented, the participants in the two vaccine groups showed similar maternal and neonatal safety profiles in both maternal age strata (Table 2). Elective terminations accounted for 86.6% (1397/1614) of termination events, followed by spontaneous abortions (10.0%, 161/1614). In total, there were 1730 delivery events with 1738 newborns, almost all of which were singleton pregnancies, with only four twin pregnancies occurred in each vaccine group. No differences were observed in the weights, gestational weeks, Apgar scores, and sex distribution of the newborns between the two vaccine groups in both maternal age strata (*p* > 0.05). The most common neonatal abnormality in both groups was abnormal weight, with the proportion being 6.1% (51/836)/7.8% (59/761) and 16.1% (10/62)/12.7% (10/79), respectively, among women with non-advanced or advanced pregnancy. Only three newborns were documented with congenital anomaly and other neonatal complications, of which two infants in the HE vaccine group were diagnosed with congenital ureter stenosis of the right kidney and heritable thalassaemia, respectively, and one male newborn in the HPV vaccine group presented with neonatal asphyxia at birth. These events were not considered to be related to vaccination by the data and safety monitoring board. A small number of pregnancy complications in focus were observed, and the differences were not statistically significant between two vaccine groups.

**Table 2.** Overall summary of pregnancy outcomes and complications.

		Non-advanced maternal age			Advanced maternal age		
		HE vaccine group	HPV vaccine group	*p*-value	HE vaccine group	HPV vaccine group	*p*-value
Termination							
	No. (%)	672/1505 (44.7)	691/1449 (47.7)	0.0979	118/179 (65.9)	133/211 (63.0)	0.5528
	Causes of termination-No. (%)						
	Elective termination	583/1505(38.7)	597/1449(41.2)	0.1718	99/179(55.3)	118/211(55.9)	0.9027
	Spontaneous abortion	66/1505(4.4)	69/1449(4.8)	0.6242	13/179(7.3)	13/211(6.2)	0.6639
	Stillbirth	13/1505(0.9)	12/1449(0.8)	0.9158	3/179(1.7)	1/211(0.5)	0.3371
	Maternal complications	10/1505(0.7)	13/1449(0.9)	0.4719	3/179(1.7)	1/211(0.5)	0.3371
Delivery							
	No. (%)	833/1505 (55.3)	758/1449 (52.3)	0.0979	61/179 (34.1)	78/211 (37.0)	0.5528
	Number of newborns*	836	761		62	79	
	Gender of newborns-No. (%)			0.8669			0.7456
	Male	459 (54.9)	421 (55.3)		37 (59.7)	45 (57.0)	
	Female	377 (45.1)	340 (44.7)		25 (40.3)	34 (43.0)	
	Median gestational week of newborns (IQR)	39.4 (38.9,40.0)	39.6 (39.0,40.0)	0.8689	39.0 (38.0,40.0)	39.1 (38.4,40.0)	0.1328
	Median weight of newborns (IQR)	3.3 (3.0,3.6)	3.3 (3.1,3.7)	0.4556	3.5 (3.1,3.7)	3.4 (3.1,3.7)	0.5286
	Median Apgar score of newborns (IQR)	10 (9,10)	10 (9,10)	0.5600	10 (10,10)	10 (9,10)	0.2422
	Neonatal abnormality-No. (%)						
	Abnormal weight	51/836 (6.1)	59/761 (7.8)	0.1928	10/62 (16.1)	10/79 (12.7)	0.5577
	Preterm birth	26/836 (3.1)	30/761 (3.9)	0.3666	6/62 (9.7)	5/79 (6.3)	0.5535
	Low Apgar score	10/836 (1.2)	6/761 (0.8)	0.4138	1/62 (1.6)	2/79 (2.5)	1.0000
	Congenital anomaly and other neonatal complications^†^	2/836 (0.2)	1/761 (0.1)	1.0000	0	0	–
Pregnancy complications in focus		17/1505(1.1)	24/1449 (1.7)	0.2212	6/179 (3.4)	4/211 (1.9)	0.5529

*Four twin pregnancies occurred in both groups, resulting in a greater number of newborns than delivery events.

^†^ In the HE vaccine group, a female infant was diagnosed with congenital ureter stenosis of the right kidney and a male infant was diagnosed with heritable thalassaemia. In the HPV vaccine group, a male newborn presented with neonatal asphyxia at birth. These events were not considered to be related to vaccination by the data and safety monitoring board.

Abbreviations: HE, hepatitis E; HPV, human papillomavirus; No., number; IQR, interquartile range.

Table 3 showed the reported adverse events of the 140 women (66 in HE vaccine group and 74 in HPV vaccine group) who were vaccinated inadvertently during pregnancy. The overall reactogenicity profiles of the two vaccines were similar. The incidence of serious adverse events (SAEs) after vaccinating during pregnancy in the two groups was comparable (*p* *= *0.1080), and none of them were judged as related to vaccination by the investigators. All the adverse reactions were mild and with severity no more than grade 2 (data not shown). The incidence of adverse reactions between the two groups showed no statistical difference (31.8% vs 35.1%, *p* = 0.6782). Local symptoms, such as pain, redness, induration, and pruritus at the injection site, occurred in both groups. Moreover, the most common systemic adverse reaction in both groups was fever (18.2% vs 20.3%, *p* = 0.7545), with all events of fever being grade 1 (axillary temperature between 37.1°C and 37.5°C). There were also no statistically significant differences in the incidences of SAE, systemic symptom, and local symptom among women who received HE vaccine during pregnancy compared with matched nonpregnant vaccinees (Table 3).

**Table 3.** Adverse events in women who were inadvertently vaccinated during pregnancy.

Classification of adverse events	HE vaccine group (*N* = 66)	HPV vaccine group (*N* = 74)	*p*-value^‡^	Matched nonpregnant HE vaccinees (*N* = 132)	*p*-value^§^
Serious adverse events after vaccinating during pregnancy *-No. (%)	3 (4.5)	9 (12.2)	0.1080	10 (7.6)	0.5499
Adverse reaction^†^-No. (%)	21 (31.8)	26 (35.1)	0.6782	46 (34.8)	0.6710
Systemic symptom	17 (25.8)	19 (25.7)	0.9912	35 (26.5)	0.9091
Fever	12 (18.2)	15 (20.3)	0.7545	23 (17.4)	0.8952
Myalgia	1 (1.5)	2 (2.7)	1.0000	5 (3.8)	0.6657
Headache	2 (3.0)	1 (1.4)	0.6017	3 (2.3)	1.0000
Fatigue	3 (4.5)	0 (0.0)	0.1022	4 (3.0)	0.6880
Allergic reaction	1 (1.5)	0 (0.0)	0.4714	2 (1.5)	1.0000
Diarrhoea	1 (1.5)	1 (1.4)	1.0000	2 (1.5)	1.0000
Cough	0 (0.0)	0 (0.0)	-	4 (3.0)	0.3034
Nausea	0 (0.0)	1 (1.4)	1.0000	1 (0.8)	1.0000
Dizziness	0 (0.0)	0 (0.0)	-	1 (0.8)	1.0000
Abdominal pain	0 (0.0)	0 (0.0)	-	1 (0.8)	1.0000
Local symptom	9 (13.6)	12 (16.2)	0.6696	18 (13.6)	1.0000
Pain	4 (6.1)	10 (13.5)	0.1423	15 (11.4)	0.2324
Redness	1 (1.5)	2 (2.7)	1.0000	0 (0.0)	0.3333
Pruritus	4 (6.1)	1 (1.4)	0.1880	4 (3.0)	0.4446
Swelling	1 (1.5)	0 (0.0)	0.4714	2 (1.5)	1.0000
Induration	1 (1.5)	4 (5.4)	0.3701	2 (1.5)	1.0000

*Serious adverse events included only those that occurred after vaccinating during pregnancy. None of severe adverse events reported was related to the vaccine.

^†^ Adverse reaction: Adverse events related to vaccine. For the HE vaccine group and the HPV vaccine group, adverse reactions included only those that occurred within 30 days after the vaccine administered during pregnancy. For matched nonpregnant HE vaccinees, adverse events were included and analysed only for the matched doses with the pregnant women group.

^‡^ *p*-value for the comparison between the HE vaccine group and the HPV vaccine group.

^§^ *p*-value for the comparison between the HE vaccine group and the matched nonpregnant HE vaccinees.

Abbreviations: HE, hepatitis E; HPV, human papillomavirus; No., number.

The incidence of each adverse pregnancy outcome was similar between the HE vaccine group and the HPV vaccine group (Supplementary Table S1). Adverse pregnancy outcome of spontaneous abortion has the highest incidence, with 5.9% and 6.4% in two groups, respectively (*p* *= *0.6056). The incidence of stillbirth was lower, at 1.3% and 1.0%, respectively (*p* *= *0.5811). The incidence of pregnancy complications in focus was also comparable (1.7% vs 2.2%, *p* *= *0.3852). As presented in Table 4, in both strata of pregnancy events with proximal or distal exposure to vaccination, the risks of each type of adverse pregnancy outcome in the HE vaccine group were comparable to that in the HPV vaccine group. The proximal exposure to HE vaccination was not associated with a significantly higher risk of abnormal foetal loss than that of HPV vaccination (OR 0.80, 95% CI 0.38–1.70), as did distal exposure (OR 1.02, 95% CI 0.76–1.37). HE vaccination exposure at either time window (proximal exposure: OR 2.46, 95% CI 0.74–8.18; distal exposure: OR 0.91, 95% CI 0.66–1.24) was also not associated with neonatal abnormality, compared with HPV vaccination. Within the HE vaccine group, significant differences were not noted between proximal and distal exposure in terms of abnormal foetal loss (OR 1.17, 95% CI 0.64–2.15), neonatal abnormality (OR 0.82, 95% CI 0.40–1.68) and pregnancy complications in focus (OR 1.34, 95% CI 0.37–4.86). And there were no differences in the occurrences of pregnancy complications among the different strata of vaccination exposure. The percentage of each pregnancy complication in focus was presented comprehensively in Supplementary Table S2. A sensitivity analysis for exposure time, which changed the definition of proximal exposure from the onset of pregnancy within 90 days post vaccination to 30 days post vaccination, was conducted (Supplementary Table S3). Similarly, increased risk of any adverse pregnancy event was not observed in women who received the HE vaccine during pregnancy or started the pregnancy within 30 days post vaccination.

**Table 4.** Association between exposure to vaccination and adverse pregnancy outcomes or pregnancy complications*.

	Proximal exposure				Distal exposure				Exposure at any time				OR^†^ (95% CI)	p-value^†^
	HE vaccine group (n=213)	HPV vaccine group (n=225)	OR (95% CI)	p-value	HE vaccine group (n=1471)	HPV vaccine group (n=1435)	OR (95% CI)	p-value	HE vaccine group (n=1684)	HPV vaccine group (n=1660)	OR (95% CI)	p-value		
Abnormal fetal loss	14 (6.6)	18 (8.0)	0.80 (0.38,1.70)	0.5668	94 (6.4)	91 (6.3)	1.02 (0.76,1.37)	0.9037	108 (6.4)	109 (6.6)	0.98 (0.74,1.29)	0.8935	1.17 (0.64,2.15)	0.6092
Spontaneous abortion	14 (6.6)	13 (5.8)	1.13 (0.51,2.53)	0.7604	65 (4.4)	69 (4.8)	0.92 (0.65,1.31)	0.6560	79 (4.7)	82 (4.9)	0.95 (0.69,1.31)	0.7585	1.67 (0.91,3.08)	0.0989
Stillbirth	0 (0.0)	1 (0.4)	NA	NA	16 (1.1)	12 (0.8)	1.31 (0.62,2.78)	0.4853	16 (1.0)	13 (0.8)	1.22 (0.58,2.55)	0.5948	NA	NA
Maternal complications	0 (0.0)	4 (1.8)	NA	NA	13 (0.9)	10 (0.7)	1.29 (0.56,2.98)	0.5478	13 (0.8)	14 (0.8)	0.93 (0.43,2.00)	0.8557	NA	NA
Neonatal abnormality	9 (4.2)	4 (1.8)	2.46 (0.74,8.18)	0.1429	80 (5.4)	86 (6.0)	0.91 (0.66,1.24)	0.5363	89 (5.3)	90 (5.4)	0.98 (0.72,1.32)	0.8880	0.82 (0.40,1.68)	0.5876
Abnormal weight	6 (2.8)	2 (0.9)	3.28 (0.64,16.82)	0.1542	54 (3.7)	66 (4.6)	0.79 (0.55,1.15)	0.2174	60 (3.6)	68 (4.1)	0.87 (0.61,1.24)	0.4394	0.81 (0.34,1.95)	0.6396
Preterm birth	1 (0.5)	2 (0.9)	0.52 (0.05,5.97)	0.6004	30 (2.0)	32 (2.2)	0.92 (0.55,1.55)	0.7637	31 (1.8)	34 (2.0)	0.91 (0.55,1.51)	0.7087	0.26 (0.03,1.95)	0.1905
Low Apgar score	1 (0.5)	2 (0.9)	0.52 (0.05,5.75)	0.5964	8 (0.5)	6 (0.4)	1.31 (0.45,3.77)	0.6196	9 (0.5)	8 (0.5)	1.11 (0.43,2.88)	0.8298	0.84 (0.11,6.58)	0.8711
Congenital anomaly and other neonatal complications	1 (0.5)	0 (0.0)	NA	NA	1 (0.1)	1 (0.1)	1.00 (0.06,16.70)	1.0000	2 (0.1)	1 (0.1)	1.97 (0.18,21.71)	0.5804	7.58 (0.78,73.66)	0.0807
Pregnancy complications in focus	3 (1.4)	5 (2.2)	0.65 (0.15,2.82)	0.5684	20 (1.4)	23 (1.6)	0.85 (0.46,1.59)	0.6191	23 (1.4)	28 (1.7)	0.82 (0.46,1.45)	0.4927	1.34 (0.37,4.86)	0.6592

*The reported measures of association are odds ratios estimated with the use of logistic regression model.

†Comparison of the risk between proximal and distal exposures was specifically drawn in the HE vaccine group.

Abbreviations: HE, hepatitis E; HPV, human papillomavirus; OR, odds ratio; NA, no answer; CI, confidence interval.

## Discussion

This post-hoc analysis showed that the HE vaccine did not have a negative effect on pregnancy relative to risks observed in HPV-vaccinated subjects. The incidences of adverse pregnancy outcomes were low and comparable between the HE vaccine receivers and HPV vaccine receivers. No potential risk was observed in vaccinated women of both the advanced or non-advanced maternal age. Additionally, there was no increased risk of abnormal foetal loss, neonatal abnormality, and pregnancy complication in women who received the HE vaccine during pregnancy or within 90 days pre-pregnancy compared with those who became pregnant at more than 90 days after vaccination. The vaccine was well tolerated during pregnancy and no vaccine-related SAE was reported.

Since licensure in 2006, over 500 million doses of HPV vaccines have been distributed and 126 countries have introduced the HPV vaccine into their national immunization programmes [10,20]. As of January 2023, Cecolin has been licensed in China, Morocco, Nepal, Thailand, and Congo (DRC) while Hecolin has been licensed only in China and Pakistan. Although HPV vaccines have not yet been recommended for use in pregnant women, accumulating evidences from clinical trials and post-marketing surveillance revealed no adverse signals after maternal HPV vaccine exposure [21–23]. Also, the Global Advisory Committee for Vaccine Safety (GACVS) has not identified any safety concerns through regular review [10]. Therefore, receiving HPV vaccine during pregnancy does not cause for alarm. As stated in the updated WHO position paper, termination of pregnancy is not indicated if vaccination was carried out inadvertently during pregnancy, and data on the safety of HPV vaccination during pregnancy are thought as reassuring [10]. Therefore, we compared the safety profile of Hecolin with that of Cecolin in the absence of the unvaccinated control population.

A large proportion of terminations were caused by elective terminations in our study, which may be closely associated with China’s birth control policy. During the first year of our study, only couples who were both from one-child families were allowed to have two children. Since November 2013, a selective two-child policy was available if either spouse was an only child, and a universal two-child policy was implemented by Chinese government after 2016 [24]. In line with the changes in fertility policy, we found that elective terminations that occurred prior to 2016 accounted for approximately 2/3 (data not shown) of all the elective terminations occurring throughout the study. Among the pregnant women, the incidence of spontaneous abortion was higher than that of stillbirth and termination caused by maternal complications. The incidence of spontaneous abortion was 5.9% in the HE vaccine group, which was similar as the prevalence of spontaneous abortion in Chinese pregnant women described in other studies [25–27]. In a retrospective study containing around 100,000 live births in southern China, the estimated percentages of low birth weight and macrosomia were about 8.7% and 3.1% between 2005 and 2017 [28]. Our study showed a resemblance, where the proportion of newborns with abnormal weight was 6.1% and 16.1% among those whose mothers were of non-advanced or advanced maternal age in the HE vaccine group, respectively. Our study showed the preterm birth rate in the HE vaccine group was 2.4%, which was lower than data from a large-scale observational study of more than 9 million Chinese pregnant women (5.9% in 2012 and 6.4% in 2018) [29]. The incidence rates of each complication during pregnancy ranged from 0.9% to 4.2% according to previous reports [30], which were similar to the overall incidence of pregnancy complications in focus (1.7%) in the HE vaccine group in our study.

There is increasing momentum to develop and implement maternal immunization to prevent specific infections to which pregnancy and newborns are susceptible. Theoretically, it is safe to receive an inactivated vaccine during pregnancy, whereas live vaccines should be contraindicated owing to the potential risk of infection to the foetus [31]. Furthermore, pregnancy is not a contraindication to the use of some recombinant sub-unit vaccines, such as the hepatitis B vaccine [32]. Several recombinant HBV vaccines approved for decades are currently in use worldwide, and no evidence of an increased risk of adverse pregnancy outcomes was observed in more than 20 years of post-marketing surveillance [33,34]. Influenza vaccine and Tdap vaccine have also been currently recommended internationally for pregnant women. HAV vaccine, HBV vaccine, serogroup B meningococcal vaccine, and pneumococcal vaccine have been recommended for specific high-risk groups of pregnancy [35,36]. No events of adverse pregnancy outcomes associated with the HE vaccine were reported by post-marketing surveillance.

As the only one commercialized HE vaccine around the world, Hecolin is of high priority for susceptible populations, including women of child-bearing age, and vaccinating women of child-bearing age has been indicated to be cost-effective from the societal perspective especially in epidemic regions [37,38]. In 2022, Médecins Sans Frontières (MSF) and South Sudan’s Ministry of Health jointly carried out two rounds of the hepatitis E vaccination campaign in Bentiu internally displaced persons camp in South Sudan’s Unity state. Around 25,000 people, including pregnant women, have received the vaccine, which was the first rolled-out vaccination in HE epidemic region [39]. To our knowledge, our study is the first one to systematically describe adverse pregnancy outcomes after exposure to HE vaccine during or around the time of pregnancy. These results also confirmed and considerably expanded on results from previous studies of the vaccine, providing additional support for the safety of the vaccine in pregnant women. Certainly, it is of great public health significance to promote clinical research and post-marketing surveillance about maternal vaccination.

This post-hoc analysis was accompanied by a number of limitations. Firstly, the long-term effects of the vaccine on infants could not be analysed because the pregnancy events were followed only up to one month after delivery. Another limitation was the small number of women vaccinated during pregnancy. It is challenging to observe rare vaccine-related adverse outcomes based on such a limited sample size. A randomized, controlled clinical trial designed specifically for pregnant women would be the “golden rule” to rule out a meaningful difference in the percentage of adverse pregnancy outcomes. In addition, this phase 3 clinical trial was designed to evaluate the efficacy against HPV infection, therefore the antibody response specific to HEV after vaccination during pregnancy was not assessed and remained to be investigated.

In conclusion, HE vaccination during or shortly before pregnancy is not associated with increased risks for both the pregnant women and pregnancy outcomes, but caution is needed until more adequate evidence is available. These data may be helpful to guide clinical practice in reducing undue concerns about the safety of vaccination during or shortly before pregnancy.

## Supplementary Material

Supplemental Material
