# Supplementary material for: Safety of hepatitis E vaccination for pregnancy: a post-hoc analysis of a randomized, double-blind, controlled phase 3 clinical trial
Source: Emerg Microbes Infect. 2023 Mar 15;12(1):2185456. doi: 10.1080/22221751.2023.2185456 (PMC10026809; doi:10.1080/22221751.2023.2185456)
Supplement: Supplemental Material [file TEMI_A_2185456_SM5056.docx]

**Caption for supplementary material**

**Supplementary Table 1. Incidence of adverse pregnancy outcomes and pregnancy complications**

**Supplementary Table 2. Detailed list of pregnancy complications in focus**

**Supplementary Table 3. Sensitivity analysis of association between exposure to vaccination and adverse pregnancy outcomes or pregnancy complications**

| **Supplementary Table 1. Incidence of adverse pregnancy outcomes and pregnancy complications^*^** | | | |
| --- | --- | --- | --- |
|  | **HE vaccine group**  **(N=1263)** | **HPV vaccine group**  **(N=1259)** | **p-value** |
| **Abnormal fetal loss-No. (%)** |  |  |  |
| Spontaneous abortion | 75 (5.9) | 81 (6.4) | 0.6056 |
| Stillbirth | 16 (1.3) | 13 (1.0) | 0.5811 |
| Maternal complications | 13 (1.0) | 14 (1.1) | 0.8401 |
| **Neonatal abnormality-No. (%)** |  |  |  |
| Abnormal weight | 59 (4.7) | 68 (5.4) | 0.4021 |
| Preterm birth | 30 (2.4) | 33 (2.6) | 0.6925 |
| Low Apgar score | 9 (0.7) | 8 (0.6) | 0.8128 |
| Congenital anomaly and other neonatal complications | 2 (0.2) | 1 (0.1) | 1.0000 |
| **Pregnancy complications in focus-No. (%)** | 22 (1.7) | 28 (2.2) | 0.3852 |
| ^*^The numerator is the number of pregnant women with adverse pregnancy events, and the denominator is the total number of pregnant women.  Abbreviations: HE, hepatitis E; HPV, human papillomavirus; No., number. | | | |

| **Supplementary Table 2. Detailed list of pregnancy complications in focus** | | |
| --- | --- | --- |
| **Pregnancy complication-No. (%)^*^** | **HE vaccine group**  **(N=1684)** | **HPV vaccine group**  **(N=1660)** |
| Ectopic pregnancy | 21 (1.2) | 23 (1.4) |
| Gestational hypertension | 2 (0.1) | 1 (0.1) |
| Preeclampsia | 0 | 2 (0.1) |
| Gestational diabetes | 0 | 2 (0.1) |
| *Calculated based on the number of pregnant events.  Abbreviations: HE, hepatitis E; HPV, human papillomavirus; No., number. | | |

| **Supplementary Table 3. Sensitivity analysis of association between exposure to vaccination and adverse pregnancy outcomes or pregnancy complications^*^** | | | | | | | | | | |
| --- | --- | --- | --- | --- | --- | --- | --- | --- | --- | --- |
|  | **Proximal exposure** | | | | **Distal exposure** | | | | **Proximal exposure vs Distal exposure^†^** | |
|  | **HE vaccine**  **(n=115)** | **HPV vaccine**  **(n=116)** | **OR**  **(95% CI)** | **P-value** | **HE vaccine (n=1569)** | **HPV vaccine (n=1544)** | **OR**  **(95% CI)** | **P-value** | **OR**  **(95% CI)** | **P-value** |
| **Abnormal fetal loss** | 7 (6.1) | 10 (8.6) | 0.68 (0.25,1.85) | 0.4537 | 101 (6.4) | 99 (6.4) | 1.01 (0.76,1.35) | 0.9319 | 1.06 (0.48,2.36) | 0.8815 |
| **Neonatal abnormality** | 3 (2.6) | 2 (1.7) | 1.50 (0.25,9.16) | 0.6611 | 86 (5.5) | 88 (5.7) | 0.96 (0.71,1.31) | 0.8102 | 0.49 (0.15,1.60) | 0.2387 |
| **Pregnancy complications in focus** | 2 (1.7) | 4 (3.4) | 0.48 (0.09,2.67) | 0.4022 | 21 (1.3) | 24 (1.6) | 0.87 (0.48,1.60) | 0.6615 | 1.69 (0.38,7.60) | 0.4941 |
| ^*^ The reported measures of association are odds ratios estimated with the use of logistic regression model. In the sensitivity analysis, proximal exposure was defined as vaccination during pregnancy or the onset of pregnancy within 30 days post any dose.  ^†^ Comparison of the risk between proximal and distal exposures was drawn in the HE vaccine group.  Abbreviations: HE, hepatitis E; HPV, human papillomavirus; No., number; OR, odds ratio; CI, confidence interval. | | | | | | | | | | |
